# Structure, Spatial and Temporal Distribution of the *Culex pipiens* Complex in Shanghai, China

**DOI:** 10.3390/ijerph13111150

**Published:** 2016-11-17

**Authors:** Qiang Gao, Chenglong Xiong, Fei Su, Hui Cao, Jianjun Zhou, Qingwu Jiang

**Affiliations:** 1 Department of Public Health Microbiology, School of Public Health, Fudan University, Shanghai 232000, China; gaoqiang110209@163.com (Q.G.); xiongchenglong@fudan.edu.cn (C.X.); 2 Key Laboratory of Public Health Safety, Ministry of Education, Fudan University, Shanghai 232000, China; 3 Department of Vectors Prevention, Center for Disease Control & Prevention, Huangpu District, Shanghai 232000, China; sufei@hpcdc.sh.cn (F.S.); caohui@hpcdc.sh.cn (H.C.)

**Keywords:** *Culex pipiens* complex, population structure, seasonality, micro-environment, DV/D ratio, *C. p. molestus*, downtown Shanghai

## Abstract

Background: *Culex pipiens molestus* was first reported in Shanghai in 2010. The population structures and seasonal distributions of *Culex pipiens* subspecies *C. p. molestus*, *Culex pipiens pallens*, and *Culex pipiens quinquefasciatus* are not well known. Methods: From late February to November 2013, we conducted daily field surveillance of mosquitoes at eight sites at two green lands and three residential areas in downtown Shanghai. Morphological comparison and DV/D ratios (DV/D is an indicator of mosquito taxonomy) were used to identify adult mosquitoes. Results: The distribution curves of the *Culex pipiens* complex members indicated seasonal fluctuations. The temperature range of 20–25 °C was the most suitable for adult activity. Micro-environmental factors may differentiate the complex population structures. Hybridization between *C. p. pallens* and *C. p. quinquefasciatus* was common and neither “DV/D = 0.40” nor “DV/D = 0.50” can distinguish these subspecies and their hybrids. *Conclusion*: the population structure of the *Culex pipiens* complex is complex and characterized by significant hybridization. Measures other than DV/D ratios are needed for the discrimination of subspecies. The *C. p. molestus* invasion might result in the transmission of novel vector-borne diseases in Shanghai.

## 1. Introduction

The *Culex pipiens* complex (Diptera: Culicidae) has been extensively studied but the taxonomic composition of the group remains unclear [1,2]. Vinogradova [1] considered the complex to include: *C. p. pipiens* with the typical, anautogenous *pipiens* form (form *C. p. pipiens*) as well as the autogenous *molestus* form (form *C. p. molestus*), *C. p. quinquefasciatus*, *C. p. pallens*, *C. torrentium* and *C. vagans*. The *C. pipiens* complex, according to Sirivanakam (1976), mainly includes the four forms *C. p. pipiens*, *C. p. molestus*, *C. p. pallens* and *C. p. quinquefasciatus* [3].

**Figure 1:**
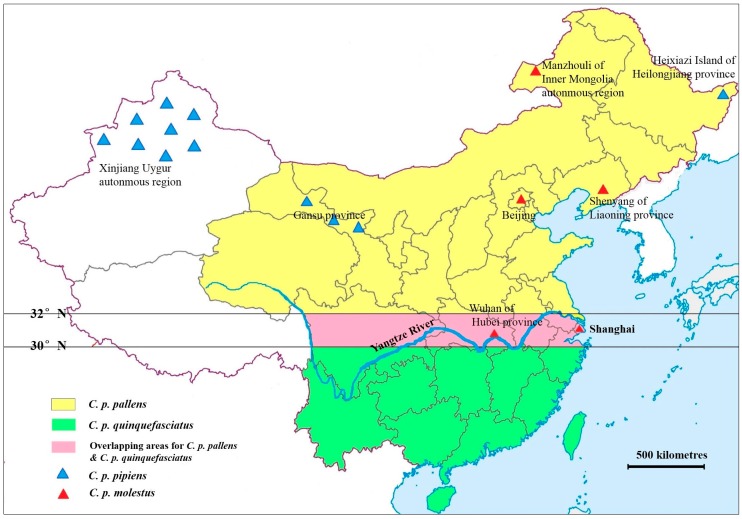
Approximate distribution of *Culex pipiens* complex mosquitoes in China (map constructed mainly from References [5,6,7,8,9,10]).

These latter four members of the *Culex pipiens* complex have been recorded in China (Figure 1) [4]. Two types of ubiquitous “house mosquitoes” (*C. p. quinquefasciatus* and *C. p. pallens*) are widely distributed in southern and northern China, respectively, with intermediates of the two found in a zone approximately between the 30° and 32° N latitude [5]. The typical *C. p. pipiens* is only found in the Xinjiang Uygur autonomous region, and autogenous *C. p. molestus* was first reported in China in the Beijing underground water system in 1992 [6]. Up until now, only five regions in China have reported the presence of *C. p. molestus*, including Beijing (1993) [6], Shenyang of Liaoning Province [7], Manzhouli of the Inner Mongolia autonomous region [8], Wuhan of Hubei Province [9] and Shanghai [10]. Shanghai is located between 30°40′ and 31°53′ N, the overlap area of *C. p. quinquefasciatus* and *C. p. pallens*, so both the forms and their hybrids are present in the region. Since *C. p. quinquefasciatus* and *C. p. pallens* females are morphologically undistinguishable, the two forms as well as their hybrids usually are classified as *C. p. pallens*. Therefore, the composition of the *C. pipiens* complex population in this area is still unclear. In Shanghai, the proportion of the autogenous *C. p. molestus* population is unknown. This subspecies was first reported by Ji-shuhong et al. in 2010, after captures were made in an underground garage catch basin [10]. Between 2013 and 2015, we also found several autogenous *C. p. molestus* breeding in the underground catch basins of downtown Shanghai.

The *C. pipiens* complex plays an important role in the transmission of several pathogens including West Nile virus (WNV), St. Louis encephalitis virus, and filarial worms [11,12,13] as well as wildlife pathogens such as avian malaria (*Plasmodium* spp.) [14]. In the past two decades, the presence of *C. pipiens* in urban areas has attracted considerable attention due to their role as the major vector of WNV in temperate regions such as Europe and North America [15,16,17,18].

In China, members of the *C. pipiens* complex have been important vectors of *Wuchereria bancrofti* [4]. In North America, *C. pipiens* is strongly associated with West Nile virus transmission. Most studies on the *C. pipiens* complex have focused on species composition or population structure, while their temporal distributions are ignored. Information about the population structure, and spatial and temporal distributions of the different *C. pipiens* subspecies, biotypes or the hybrids is lacking in most areas of China due to the lack of sensitive and specific high-throughput screening of large sample sizes. To provide a rationale for WNV preventions in Shanghai, we developed a detailed investigation on the population structure and distribution of the *C. pipiens* complex, as well as their variances caused by micro-environmental factors.

## 2. Materials and Methods

### 2.1. Locations and Characteristics of Study Areas

Fieldwork was carried out at the central area of Shanghai Municipality, located in east China (31°13′ N, 121°27′ E and 3.5 m above sea level), at two green lands (People’s Park and People’s Square) and three different residential environments. People’s Park and People’s Square are located in the center of Shanghai. The area is surrounded by busy roads, and the busiest People’s Square Subway Station The exchange center for the subway Lines 1, 2, and 8 is located underground.

**Figure 2:**
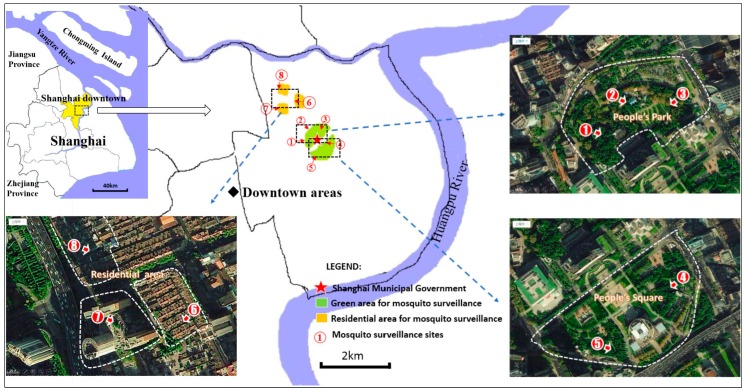
Regions and sites for mosquito surveillance in downtown Shanghai (Huangpu district).

Mosquito surveillance sites 1–3 were set in People’s Park located south of Nanjing Lu Road. The sites represented the west, middle and east area. The east and west parts have plentiful tall-vegetation and landscape architectures, including pavilions, corridors, artificial hills, lotus pond and pergolas, tourists can have a rest, play chess, or have a picnic on the stone tables and stone stools in the jungle of the area. With less vegetation, a small children’s playground with several amusement facilities, a teahouse, an outdoor theater, and a dance hall can be found in the vast middle area. Two mosquito surveillance sites (sites 4 and 5) were set at the east and west areas of the People’s Square. There is an artificial dovecot in the west part of the area, thousands of doves are active in this part. Three mosquito surveillance sites (sites 6–8) were located in residential areas in downtown Shanghai (Figure 2 and Table 1). Daily meteorological data was obtained from Shanghai Meteorological Center.

### 2.2. Study Design

From late February to November 2013 (224 days), adult mosquito surveillance occurred daily at the eight aboveground sites (Figure 2). Mosquitoes were captured using a CO_2_-light trap and preserved in 75% ethanol. Compressed CO_2_ gas (5 kg/cylinder) and trapping-bait (product simulating body secretions, produced by Bat King, Shanghai, China) were replaced every five and 20 days, respectively.

### 2.3. Identification and Classification of Captured Mosquitoes

With reduced thoracic chaetotaxy (the presence of specific setae and/or scales), wild-caught females of *C. pipiens* complex subspecies were morphologically indistinguishable [19] and were discarded. Adult males were dissected and classified according to the morphological characters of their genitalia. There are four forms of *C. pipiens* complex in downtown Shanghai. Phallosome differences were apparent among typical *C. p. molestus*, *C. p. quinquefasciatus* and *C. p. pallens*, though there was a small percentage of atypical species as well as putative hybrids. We considered the typical and atypical subtypes as one separate form and the hybrids as another separate form.

DV is the extension of the ventral arm laterally of its intersection with the dorsal arm and D is the distance between the two intersections of the dorsal and ventral arms. Calculation of the DV/D ratio is done according to Barr [20]. Median, “Mean ± STD (standard deviation)” and interval from minimum to maximum were the indicators used to describe the distribution and dispersion of DV/D ratio among the four *C. pipiens* forms. DV measurements were recorded and their means calculated. Measurements were made by using an ocular micrometer at 100 diameters magnification, and were estimated to the nearest half of a scale division.

### 2.4. Statistical Analysis

Data were analyzed using the SPSS version 11.5 (SPSS Inc., Chicago, IL, USA) statistical package. The relative abundance of mosquito forms was expressed as “mosquito counts” instead of “mosquito density” because the size of male samples was relatively small. The variance between the proportions of different forms of *C. pipiens* complex was tested by chi-square analysis. Differences of DV/D values among the four forms were compared by nonparametric analysis of variance (Kruskal-Wallis test) and the Nemenyi test for post hoc testing.

## 3. Results

### 3.1. Population Structure

**Figure 3:**
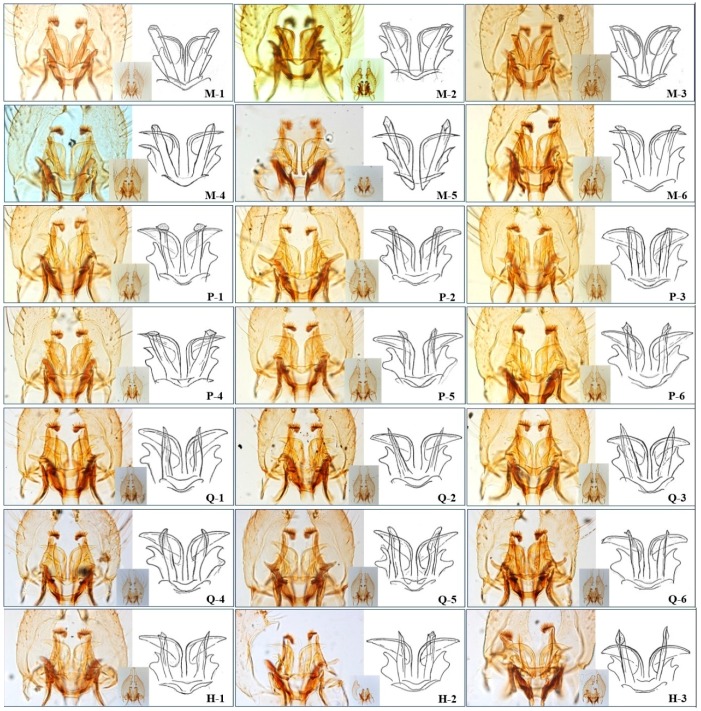
Genitalia (dorsal view, post-rotation) of male *Culex pipiens* complex samples collected in downtown Shanghai, China. Typical *C. p. molestus* (M-1, 2, 3): the dorsal arms are divergent and broad, and their tips are thick and blunt, the ventral arms are narrow, short and sharply bent laterally. Atypical *C. p. molestus* (M-4, 5, 6): the ventral arms are longer than the typical ones, and the DV/D ratio increases accordingly. Typical *C. p. pallens* (P-1, 2, 3): the dorsal arms are divergent and broad, and their tips are thick and bluntly round (or truncate), the ventral arms are broad, long and extend caudolaterally (the same as *C. p. quinquefasciatus*). Atypical *C. p. pallens* (P-4, 5, 6): the ventral arms are narrower (P-4), or the cross-section of the dorsal arms’ tips are irregular (P-5, 6). Typical *C. p. quinquefasciatus* (Q-1, 2, 3): the dorsal arms are parallel (or divergent, Q-2, 3) and narrow, and their tips are markedly pointed, the ventral arms are broad, long and extend caudolaterally. Atypical *C. p. quinquefasciatus* (P-4, 5, 6): the tips of the dorsal arms are “thumb-shaped” (Q-4), or the dorsal arms are a little bit thicker (Q-5) or flat (Q-6). Hybrids of *pallens* and *quinquefasciatus* (H-1, 2, 3): morphological character of the dorsal arms is between *C. p. pallens* and *C. p. quinquefasciatus*.

A total of 23,919 adult mosquitoes were captured at the eight sampling sites, and the collections included four mosquito species predominated by the *C. pipiens* complex (*n* = 12,334; 51.57% of total); the other three species were *Aedes albopictus*, *Culex tritaeniorhynchus* and *Anopheles sinensis*. A total of 735 male *Culex pipiens* complex mosquitoes (5.96%) were dissected, of which 721 (5.85%) were morphologically identical to their type specimens of subspecies or forms (Figure 3).

**Figure 4:**
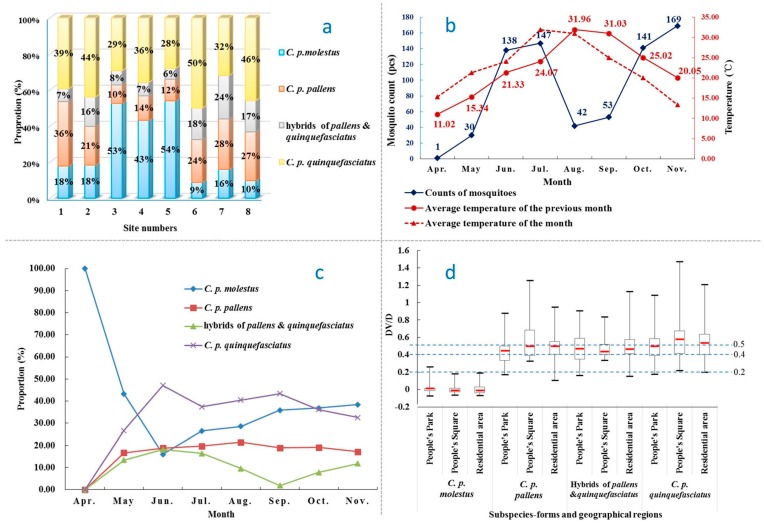
Characteristics of the structure, spatial and temporal distribution of *Culex pipiens* complex in downtown Shanghai, China. (**a**) Proportion of the four forms of *Culex pipiens* complex at the eight sites in downtown Shanghai; (**b**) Correlation between temperature and counts of *Culex pipiens* complex mosquitoes in downtown Shanghai; (**c**) Seasonal fluctuation of *Culex pipiens* complex members in downtown Shanghai; (**d**) DV/D ratio of *Culex pipiens* complex members in downtown Shanghai.

Among the four forms, *C. p. quinquefasciatus* (*n* = 274, 38.00%) and *C. p. molestus* (*n* = 223, 30.93%) were the most dominant subspecies; both were significantly more than *C. p. pallens* (*n* = 135, 18.72%) (38.00% vs. 18.72%, χ^2^ = 28.775, *p* < 0.001; 30.93% vs. 18.72%, χ^2^ = 65.943, *p* < 0.001). The hybrid form of *pallens* and *quinquefasciatus* was captured in lesser numbers (*n* = 89, 12.34%), and the ratio of the four forms was approximately 4:3:2:1 (Table 2). The population structure varies among surveillance sites. *C. p. molestus* was abundant in sites 3–5 (52.56%, 43.21% and 54.00%, respectively), and was much less common in the other five sites, especially the residential area sites 6–8 (8.75%, 16.00% and 9.72%, respectively). The distribution of *C. p. quinquefasciatus* and *C. p. pallens* was relatively similar among the eight sites, although slightly less in sites 3–5 in contrast to the high proportion of *C. p. molestus* (Figure 4a).

Differences in micro-environments might have influenced the population structure of the *C. pipiens* complex. Sites 1–3 at People’s Park are examples: even though they were at nearby locations within the same park, the three sites displayed distinct population structures (see Figure 4a). The mosquito population size in site 3 was much greater than at sites 1 and 2 (*n* = 215 vs. *n* = 28, χ^2^ = 223.561, *p* < 0.001; *n* = 215 vs. *n* = 98, χ^2^ = 80.832, *p* < 0.001); second, the proportion of *C. p. molestus* at site 3 was much larger than those on sites 1 and 2 (52.56% vs. 17.86%, χ^2^ = 11.942, *p* = 0.001; 52.56% vs. 18.37%, χ^2^ = 32.336, *p* < 0.001). Finally, the proportion of *C. p. pallens* and *C. p. quinquefasciatus* in the three sites differed (Table 2).

### 3.2. Seasonal Fluctuation

Mosquito counts increased rapidly as mean daily temperatures rose. Counts peaked in June and July (*n* = 138 and 147). As the temperature continued to rise in August and September, mosquito counts declined sharply (*n* = 42 and 53), and then peaked again in October and November (*n* = 141 and 169) as the temperatures declined. There existed an obvious relationship between temperature and the mosquito density, but the impact of the temperature on mosquitoes had an approximate one-month lag time. The most suitable temperature interval for the *C. pipiens* complex ranged between 20 and 25 °C (Figure 4b).

The proportion of *C. pipiens* complex members also exhibited distinct seasonal variation. During the low-temperature months (April, May, and November), *C. p. molestus* dominated the complex composition and peaked in November (*n* = 65, Proportion = 38.46%). In contrast, *C. p. quinquefasciatus* dominated during the high-temperature months (June to September), and peaked in June (*n* = 65, proportion = 47.10%). Population levels of *C. p. pallens* were relatively stable during the study period (Figure 4c).

### 3.3. DV/D Ratio

The DV/D ratio of *C. p. molestus* was the smallest among the four subspecies (−0.012 vs. 0.479, 0.469, and 0.500 for median; *p* < 0.01); however, its maximum of 0.258 also exceeded 0.20, which is the criteria for the classification of *C. p. pipiens* or *molestus*. Although the DV/D ratio of *C. p. pallens* was significantly smaller than that of *C. p. quinquefasciatus* (0.479 vs. 0.500 of median, Nemenyi test, *p* = 0.04), the difference was quite small. There was no significant difference between hybrids and *C. p. pallens* or *C. p. quinquefasicatus* (Table 3).

The median value and most of *C. p. molestus*’ DV/D ratios were below 0.2; however, neither 0.4 nor 0.5 could accurately separate the forms of *C. p. pallens*, *C. p. quinquefasciatus* and the hybrids (Figure 4d).

## 4. Discussion

The distribution of the *C. pipiens* complex, and in particular of the *C. p. molestus* subspecies, is of great importance in Shanghai as well as in the rest of China because of the increased number of underground mosquito breeding places in towns [1]. In Shanghai, *C. p. molestus* had never been reported before 2010, when Ji-shuhong et al. first reported the autogenous form of *C. pipiens* underground in the Yangpu district [10]. Until recently, only five regions in China had reported the presence of *C. p. molestus*. This subspecies was mostly found breeding in underground environments. Shanghai appears to be the only place in China where *C. p. molestus* is breeding in both half-closed underground garages and the open aboveground environment [10]. In a recent study (2013 to 2015), we confirmed that thousands of autogenous *C. pipiens* larvae existed in catch basins of several underground garages and, *C. p. molestus* larvae bred in an aboveground surface sewer of People’s Square (unpublished). Unfortunately, data on *C. pipiens* dispersal are limited. Most documents relate to *C. p. quinquefasciatus* which was probably introduced to the Hawaii Islands in the 18th century [21]. *C. p. quinquefasciatus* is easily transported by aircraft or boats whereas, under similar conditions, *C. p. molestus* is much rarer. Vinogradova [1] inferred that the chances for a successive introduction of *C. p. molestus* are small in the temperate belt, since (i) their manner of reproduction in closed underground habitats makes it unlikely that they will be transported by a vehicle and (ii) climatic conditions restrict the periods when mosquitoes can disperse from their habitats. If *C. p. molestus* mosquito transportation does occur, the female(s) could encounter difficulties in finding suitable underground habitats for oviposition in the new environment. Waves of founder mosquitoes are likely to be repeatedly transported before a new stable local population is established.

Generally, autogenous *C. p. molestus* is associated with breeding sites that provide only limited access and egress for these insects [22]. However, the status of *C. p. molestus* in downtown Shanghai appears to differ from the norm. Prior to this study, we estimated that there would be few adult *C. p. molestus* active aboveground and these would originate from the abundant underground garages or urban basements of Shanghai. However, the proportion of *C. p. molestus* in the open environment was as high as 52.56% (site 2). One explanation is that *C. p. molestus* has now adapted to the urban aboveground environment in Shanghai. While thousands of adult *C. p. molestus* leave their underground habitats and return to the underground for oviposition, a small proportion may have located suitable habitats for oviposition aboveground. This was verified when we found *C. p. molestus* larvae in an aboveground surface sewer of People’s Square. Regardless of the types of habitats they would prefer for oviposition, a high proportion of adult *C. p. molestus* in the open environment would minimize the dispersal constraints mentioned above, and greatly facilitate dispersal. *C. p. molestus* in Shanghai could soon be introduced to the surrounding areas or more distant regions by vehicles, boats and aircraft. An alternative interpretation is that the form *C. p. molestus* in our study is actually the form *C. p. pipiens*, which is anautogenous, eurygamous (only mating in outdoor swarms) and capable of diapause [19,23]. Vinogradova noted that both forms occur in sympatry almost throughout the entire area of distribution. In this study, if specimens of the form *C. p. pipiens* are included, they could not be separated from *C. p. molestus* morphologically in this study, and an appropriate molecular approach may be needed for further analysis [24]. In any event, the proportion of the form *C. p. molestus* (or the form *C. p. pipiens*) is fairly high in the aboveground environment. This is not good news for either Shanghai or for China because it could greatly increase the risk of transmission of WNV and other diseases vectored by the *C. pipiens* complex.

*C. pipiens* complex mosquitoes are difficult to classify by simple methods and many forms are routinely determined to be *C. p. pallens*. This is usually inaccurate, since the proportion of *C. p. pallens* is smaller than both *C. p. quinquefasciatus* and *C. p. molestus*. In addition, there is often a proportion of hybrid forms. As an approach for morphological classification, the DV/D ratio was developed from the D/V ratio. The latter is defined as a ratio of distances between the dorsal and ventral arms, which is also a method of distinguishing individuals of *C. pipiens* that was suggested by Sundararaman (1949) [25] and later modified by Barr (1957) [20] to give “somewhat more objective measurements”. The method has since been improved to result in the well-known DV/D ratio. The DV/D ratio remains the most reliable method for distinguishing adult *C. p. pipiens* and *C. p. quinquefasciatus* (“<0.20” = *pipiens*) [26]. In this study, there was no need to calculate DV/D ratios to separate *C. p. pipiens* and *C. p. quinquefasciatus*, since the dorsal and ventral arms and lateral plate are distinctive enough to distinguish the males of the two subspecies. The DV/D ratio is useful for distinguishing parental and hybrid forms in zones of introgression and establishing the temporal and geographic extent of hybridization. It works well in the United States for differentiating the subspecies and for detecting the presence of hybrids [20,27]. The DV/D ratio was also used to establish the existence of a continuous, self-maintaining intermediate population in Memphis, Tennessee [28]. Barr suggested that 0.40 should be the criteria to separate *C. p. quinquefasciatus* from other subspecies (“>0.40” = *quinquefasciatus*), but Jakob et al. suggested that 0.50 should be the lowest DV/D value denoting specimens as *quinquefasciatus* [26]. Researchers in China and other Asian countries suggest that the DV/D ratio is a poor indicator for mosquito classification, since the values of DV/D vary greatly according to latitude, season, and even use of the technique [29,30,31,32]. In this study, neither DV/D = 0.40 nor DV/D = 0.50 provided a good criterion to differentiate *C. p. quinquefasciatus* and *C. p. pallens* or the hybrids, since the values of DV/D among the three forms overlapped in the 0.40–0.50 interval. The dorsal arms of most *C. p. quinquefasciatus* are divergent with tips pointed, which is different from the classical definition of “parallel and pointed”, and greatly different from the atypical or hybrid examples. We therefore classified both the “divergent and pointed” and “parallel and pointed” forms as typical *C. p. quinquefasciatus*. Divergent dorsal arms resulted in relatively low DV/D ratio values. The median value of the DV/D ratio of *C. p. quinquefasciatus* in People’s Park was 0.50, and the mean DV/D ratio was 0.539.

Urbanization is one of the major human activities impacting biodiversity. Human intervention creates highly heterogeneous environments for invertebrates, in which many biotic and abiotic factors could affect the community structure, impacting species richness and the species composition [33]. These factors may occur at different spatial scales, from the macro-environment to the micro-environment [34,35,36]. Eight field sites for mosquito surveillance, with the average distance of less than 600 m between each other, were micro-environments within the urban area. The effect of the micro-environment on the richness (density) and the complex composition (population structure) is obvious in this study, especially for *C. p. molestus*. The factors influencing the mosquito population structure and density include types of adult mosquito habitats, types and number of breeding sites, and blood meal sources. In our understanding, the most important factor might be the types and numbers of breeding sites. Generally, *C. p. molestus* prefer breeding sites that provide only limited access and egress such as underground garages or basements. However, the eight sites are all surrounded with tall buildings that have many underground garages in the urban center. A plausible explanation is that *C. p. molestus* can oviposite in at least one type of aboveground breeding site, and such breeding sites are widely distributed at sites 3–5. The finding of an aboveground breeding site in People’s Square during the summer of 2015 supports this hypothesis.

Seasonal progressions and resulting changes (e.g., temperature and photo period) are other essential external factors greatly affecting mosquito population dynamics. In the relatively cold April, only male *C. p. molestus* were captured. This is not difficult to interpret since *C. p. molestus* can survive and breed underground without diapause while development of other forms decreases dramatically during the winter with significant activity only resuming in the spring. *C. p. quinquefasciatus* also forgoes diapause [37], but its ability to adapt to both low temperature and underground habitats is apparently less than *C. p. molestus*. The density of the *C. pipiens* complex is closely related to the temperature, but the effect of temperature is delayed for approximately one month. This lag phase can provide an excellent time window for mosquito control.

## 5. Conclusions

The population structure of *Culex pipiens* in Shanghai is complex and includes a significant amount of hybridization. *C. p. quinquefasciatus*, *C. p. pallens* and *C. p. molestus* coexist in Shanghai, producing a fairly high proportion of hybrids of *pallens* and *quinquefasciatus* and many morphologically atypical forms of the *C. pipiens* complex. In Shanghai, the *C. p. molestus* population is increasing and its habitats are no longer confined to the underground areas. The increasing number of *C. p. molestus* and its adaptation to aboveground habitats may facilitate its dispersal to other areas around China and a larger population might result in the transmission of novel vector-borne diseases. The DV/D ratio could not be used to differentiate the *C. pipiens* complex mosquitoes in Shanghai, since the values of the DV/D ratio overlapped in the three forms of the complex. The most practical method for classification is based on the morphological characters of male genitalia. The micro-environment and temperature can significantly affect the complex population structures.

## Figures and Tables

**Table 1:** Geographical information of the eight mosquito surveillance sites.

Site Numbers	Area	Environmental Types	Coordinates
1	People’s Park	Green land (Park)	31°13′59.72″ N 121°28′02.06″ E
2	People’s Park	Green land (Park)	31°14′03.10″ N 121°28′06.26″ E
3	People’s Park	Green land (Park)	31°14′02.48″ N 121°28′13.02″ E
4	People’s Square	Green land	31°13′53.01″ N 121°28′19.12″ E
5	People’s Square	Green land	31°13′47.13″ N 121°28′09.01″ E
6	Residential area	Old types “Shikumen”	31°14′11.15″ N 121°27′48.74″ E
7	Residential area	High-rise residential	31°14′11.18″ N 121°27′45.45″ E
8	Residential area	Old Villa	31°14′15.83″ N 121°27′42.76″ E

**Table 2:** Composition and distribution (Counts/pcs, proportion/%) of *Culex pipiens* complex mosquitoes in downtown Shanghai.

Site Numbers	*C. p. molestus*	*C. p. pallens*	Hybrids of *pallens* & *quinquefasciatus*	*C. p. quinquefasciatus*	Sum
1	5 (17.86)	10 (35.71)	2 (7.14)	11 (39.29)	28
2	18 (18.37)	21 (21.43)	16 (16.33)	43 (43.88)	98
3	113 (52.56)	22 (10.23)	17 (7.91)	63 (29.30)	215
4	35 (43.21)	11 (13.58)	6 (7.41)	29 (35.80)	81
5	27 (54.00)	6 (12.00)	3 (6.00)	14 (28.00)	50
6	7 (8.75)	19 (23.75)	14 (17.50)	40 (50.00)	80
7	4 (16.00)	7 (28.00)	6 (24.00)	8 (32.00)	25
8	14 (9.72)	39 (27.08)	25 (17.36)	66 (45.83)	144
People’s Park (1–3)	136 (39.88)	53 (15.54)	35 (10.26)	117 (34.31)	341
People’s Square (4, 5)	62 (47.33)	17 (12.98)	9 (6.87)	43 (32.82)	131
Residential (6–8)	25 (10.04)	65 (26.10)	45 (18.07)	114 (45.78)	249
Total	223 (30.93)	135 (18.72)	89 (12.34)	274 (38.00)	721

**Table 3:** DV/D ratios of *Culex pipiens* complex members in Shanghai downtown, China.

Forms	*n*	Median	Mean ± STD	Intervals
*C. p. molestus*	223	−0.012 ^a^	0.004 ± 0.048	−0.079–0.258
*C. p. pallens*	135	0.479 ^b^	0.486 ± 0.168	0.100–1.250
Hybrids of *pallens* and *quinquefasciatus*	89	0.469 ^b,c^	0.490 ± 0.180	0.150–1.125
*C. p. quinquefasciatus*	274	0.500 ^c^	0.539 ± 0.195	0.171–1.467

^a,b,c^: Nemenyi test for post hoc comparison between groups, no significant difference between the same letters, but there are significant differences between different letters, like ^a^ and ^b^, ^b^ and ^c^, as well as ^a^ and ^c^, *p* < 0.05.
